# Drought Intensity, Timing, and Reproductive Strategy Drive Submerged Macrophyte Resilience

**DOI:** 10.3390/plants15060943

**Published:** 2026-03-19

**Authors:** Ying He, Peizhong Liu, Chengxiang Zhang, Zijian Wang, Xiaobo Zhang, Kaidi Guo, Yangsirui Zhang, Jialin Lei, Jiaying Zhou, Qing Zeng, Cai Lu, Ting Lei, Li Wen, Guangchun Lei

**Affiliations:** 1 Centre for East Asian-Australasian Flyway Studies, Beijing Forestry University, Beijing 100083, China; abbyisxyz33@bjfu.edu.cn (Y.H.); peizhongliu@bjfu.edu.cn (P.L.);; 2 College of Ecological Engineering, Shandong University of Ecology and Environment, Weifang 261108, China; 3 School of Ecology and Nature Conservation, Beijing Forestry University, Beijing 100083, China; 4 Hebei Engineering Research Center for Ecological Restoration of Seaward Rivers and Coastal Waters, Hebei University of Environmental Engineering, Qinhuangdao 066102, China; 5 National Forestry and Grassland Administration Forest and Grassland Survey and Planning Institute, Beijing 100013, China; 6 Shaoguan Municipal Ecological Environment Monitoring Station, Shaoguan 512000, China; 7 Birdsdata Technology (Beijing) Co., Ltd., Beijing 100083, China; 8 National Field Scientific Observation and Research Station of Dongting Lake Wetland Ecosystem, Yueyang 415904, China; 9 Science and Insights Division, Department of Climate Change, Energy, the Environment and Water, Parramatta, NSW 2150, Australia; 10 Department of Earth and Environmental Sciences, Macquarie University, Sydney, NSW 2109, Australia

**Keywords:** submerged macrophytes, drought resilience, reproductive strategy, seed bank, clonal propagation, hydrological variability

## Abstract

Extreme droughts are projected to become more frequent and severe under climate change, posing significant risks to wetland ecosystems and submerged macrophyte communities. We combined field surveys in West Dongting Lake, China, combined with controlled greenhouse experiments to examine how drought intensity (expressed as contrasting soil moisture conditions during drought) and drought timing affect submerged macrophyte species richness, biomass, as well as resilience, mediated through species response in their reproductive strategies. Field observations revealed a sharp decline in clonal species (*Hydrilla verticillata*, *Ceratophyllum demersum*, *Vallisneria spinulosa*) following an extreme drought, while the sexual species *Najas marina* emerged as dominant. Greenhouse experiments confirmed these patterns and elucidated underlying mechanisms: extreme drought suppressed biomass, leaf area, and seedling re-germination in clonal species, whereas *N. marina* maintained regeneration via a persistent soil seed bank. Moderate drought enhanced leaf area, consistent with the intermediate disturbance hypothesis, while early drawdowns were most detrimental to growth. Species-specific responses highlight the role of reproductive strategy in drought resilience. These findings underscore the need for climate-adaptive water-level management, including limiting early drawdowns, mitigating extreme drought, and conserving seed banks to sustain biodiversity and ecosystem function under increasing hydroclimatic variability.

## 1. Introduction

Over the past fifty years, substantial evidence has shown that submerged macrophytes have gradually disappeared from shallow lakes [1,2,3]. Many natural and anthropogenic factors contribute to the disappearance of submerged macrophytes, including eutrophication [4,5], changes in hydrological regimes [6,7], and invasive species [8,9]. In addition, extreme weather events, also known as extreme climate events [10], are increasingly recognized as one of the major threats to submerged vegetation in shallow lentic freshwater ecosystems [1,11].

Extreme droughts driven by global warming constitute one of the most consequential forms of extreme climate events [12]. Drought—prolonged periods of markedly reduced precipitation—is a pervasive [13], high-impact disturbance across terrestrial and wetland ecosystems worldwide [14]. Numerous previous studies have shown that in shallow water bodies, climate warming can influence the distribution and coverage of submerged macrophytes [15,16,17] by altering nutrient dynamics through both internal and external loading [18], and by reshaping trophic structures and community dynamics [19,20]. In addition, severe climate warming can lead to rapid water level declines or even complete desiccation in shallow lakes, directly causing large-scale mortality of submerged macrophytes [21].

In recent years, the middle reaches of the Yangtze River, China, have experienced frequent extreme weather events. In 2022, the region suffered an exceptionally severe and prolonged drought lasting over 100 days [22]. A striking manifestation of this crisis occurred on 23 September 2022, when the water level of Poyang Lake—the largest freshwater lake in China—dropped to 7.1 m (Wusong zero baseline elevation), the lowest level recorded since 1951 [23]. As a neighboring system with highly similar climatic and hydrological characteristics, Dongting Lake (the second-largest freshwater lake in China) was similarly affected. In West Dongting Lake, long-term hydrological records from the Xiaohezui station (2013–2021)—the nearest station to this area—confirm the exceptional nature of the 2022 event. Starting from 12 July 2022, daily water levels dropped below the recorded minima of the previous nine years for the same period and remained at these historical lows for the remainder of the year (data sourced from http://yzt.hnswkcj.com:9090/#/ (accessed on 1 October 2024). Consequently, the western part of the lake, which lies at a relatively higher elevation—including areas such as Qingshan Lake, Beiwa, and Swan Lake—completely dried up. To date, research on the ecological impacts of the 2022 extreme drought in the Dongting Lake region remains limited. Existing studies have primarily focused on selected components such as waterbirds [24], zooplankton communities [25], and the intensification of eutrophication [26], while the responses of aquatic plants—particularly submerged macrophytes—have received little attention. Moreover, there is a notable lack of comparative analyses before and after the drought, as well as systematic experimental studies simulating drought conditions.

This study integrates field investigations and controlled indoor experiments to systematically examine the effects of extreme drought on submerged macrophytes in the West Dongting Lake wetland. Field surveys revealed sharp declines in clonal species, including *Hydrilla verticillata*, *Ceratophyllum demersum*, *Vallisneria spinulosa*, and the unexpected dominance of *Najas marina* post-drought, suggesting that reproductive strategy may influence resilience. These observations provided the rationale for our greenhouse experiment, which tested how drought intensity and drawdown timing interact to shape regeneration strategies. The research aims to test the following hypotheses:

(1) Extreme drought intensity will severely reduce biomass, leaf area, and seedling recruitment, particularly in clonal species;
(2) Sexual species with soil seed banks will exhibit greater resilience to drought than clonal species;
(3) Moderate drought will enhance certain traits, such as leaf area, consistent with the intermediate disturbance hypothesis;
(4) Early drawdowns will have stronger negative effects on growth and regeneration than late drawdowns.

## 2. Results

### 2.1. Submerged Macrophytes Distribution in West Dongting Lake

**Figure 1:**
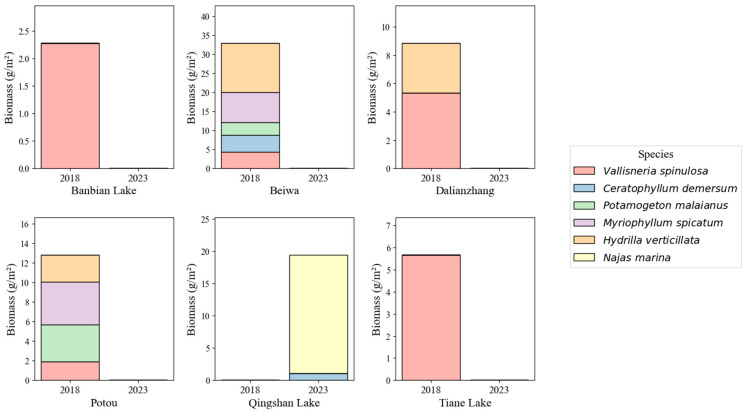
Biomass and species composition of submerged macrophytes in six lake regions of West Dongting Lake in 2018 and 2023. Each panel represents one lake region, with stacked bar charts showing the mean biomass (g/m^2^) of six submerged macrophyte species in the two survey years. Colors denote different species, as indicated in the legend. Data are averaged across all sampling points within each lake region and year. Note that extremely low biomass values (<0.10 g/m^2^) in some regions are difficult to visually distinguish due to the common scale used across lakes.

Significant differences in the community structure and biomass of submerged macrophytes were observed across different regions between 2018 and 2023. In 2018, several regions exhibited relatively high species richness and biomass, with Beiwa and Potou being the most representative regions. In Beiwa, *H. verticillata* had the highest mean biomass, reaching up to 27.02 g/m^2^. Additionally, other species such as *V. spinulosa*, *Myriophyllum spicatum*, and *C. demersum* were frequently recorded. At Potou, a multi-species assemblage dominated by *Potamogeton malaianus*, *Myriophyllum spicatum*, and *H. verticillata* was observed, indicating a relatively stable community structure. By 2023, both species richness and biomass of submerged macrophytes had declined markedly in most regions. In all sampled plots at Beiwa and Potou, only a trace amount of *V. spinulosa* (0.22 g/m^2^) remained in Beiwa, while no other species were detected. A similar pattern was observed in Banbian Lake and Tiane Swan Lake: the previously dominant *V. spinulosa* had almost disappeared, with only trace biomass (<0.10 g/m^2^) detected in a few plots—levels so low that they are barely visible in Figure 1.

Qingshan Lake displayed a contrasting pattern. While no submerged macrophytes were recorded there in 2018, both *N. marina* and *C. demersum* emerged rapidly in several plots in 2023 and became dominant species, with the biomass of *N. marina* peaking at 69.47 g/m^2^ in some locations (Figure 1).

### 2.2. Responses of Submerged Macrophyte to Drought Under Experiments

Under extreme drought conditions, regeneration was almost completely suppressed for clonal species (*H. verticillata*, *C. demersum*, and *V. spinulosa*). Biomass and leaf area were near zero, and no seedlings re-germinated for these species. In contrast, *N. marina* exhibited remarkable resilience, maintaining measurable regeneration and leaf development even under severe desiccation, although at reduced levels compared to the control.

All species showed some recovery under moderate drought, but performance varied strongly with drawdown timing: delayed drawdown consistently produced the highest biomass and leaf area among clonal species, followed by normal drawdown, while early drawdown resulted in minimal growth or regeneration failure. For seedling numbers, delayed drawdown also supported the greatest re-germination, whereas early drawdown sharply reduced survival.

*N. marina* remained comparatively stable across all timing treatments, reinforcing its drought tolerance and seed-bank advantage.

The control group exhibited the highest values for biomass, leaf area, and seedling numbers across all species, highlighting the strong suppressive effect of drought intensity and timing on submerged macrophyte growth.

Overall, these results demonstrate that extreme drought eliminates clonal species, whereas sexual reproduction enables persistence under severe stress, and that drawdown timing modulates recovery under moderate drought, with delayed exposure being most favorable.

### 2.3. Response of Submerged Macrophyte to Water Drawdown

Across all traits, drought treatments had strong and consistent effects, with extreme intensity causing severe reductions in biomass, leaf area, and seedling re-germination. Moderate intensity often maintained or even enhanced growth, particularly for leaf area. Timing effects were most pronounced for biomass and leaf area, where early timing generally reduced growth, while seedling re-germination showed weaker timing effects. Species-level variation was evident for all traits, highlighting differential resilience among taxa. These patterns suggest that intensity is the dominant driver of plant responses, with timing and species identity playing secondary but important roles.

#### 2.3.1. Drought Effects on Measured Traits

**Figure 2:**
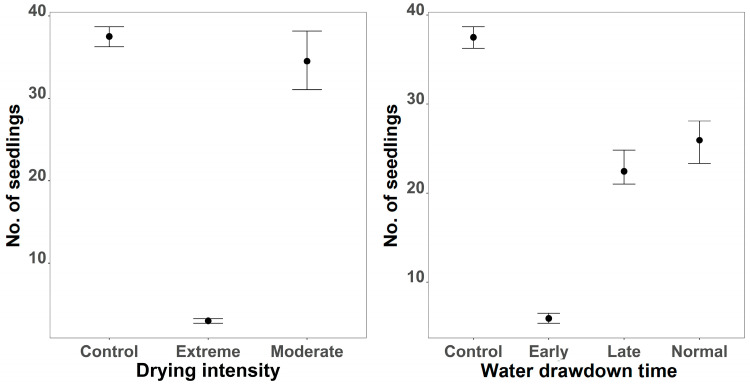
Effects of drought intensity (**left**) and water drawdown regime (**right**) on the number of re-geminated seedling. Points represent posterior means, and error bars indicate 95% credible intervals based on 10,000 posterior draws of the final GLMM model.

Seedling re-germination was strongly affected by treatment intensity, with weaker and more uncertain effects of timing (Figure 2). Extreme intensity significantly reduced seedling numbers compared to control (Estimate = −2.55, 95% CI: −4.97 to −0.12), and the credible interval excluded zero, indicating strong evidence for a negative effect. Marginal plots showed mean seedling counts near 37 under control conditions, declining to approximately 2 under extreme intensity, while moderate intensity maintained relatively high counts (~33), similar to control (Figure 2, left). Timing effects were less pronounced: early timing reduced seedling numbers (Estimate = −1.88, 95% CI: −4.70 to 0.77), but credible intervals overlapped zero. Late and normal timings showed intermediate values (~22 and ~26 seedlings) with wide intervals, suggesting weaker evidence for timing effects (Figure 2, right). Species-level variation was moderate (sd = 0.72, 95% CI: 0.04 to 2.53), and the shape parameter (0.42, 95% CI: 0.28 to 0.59) confirmed overdispersion typical of count data.

**Figure 3:**
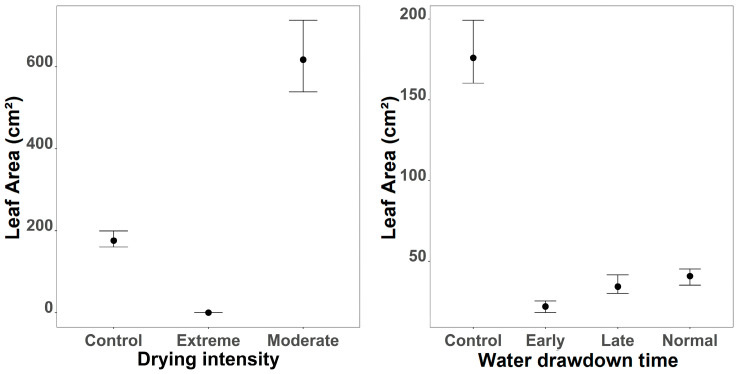
Effects of drought intensity (**left**) and water drawdown regime (**right**) on the mean leaf area per plant. Points represent posterior means, and error bars indicate 95% credible intervals based on 10,000 posterior draws of the final GLMM model.

Leaf area was most strongly influenced by intensity, with additional effects of timing and their interaction (Figure 3). Extreme intensity caused a dramatic reduction compared to control (Estimate = −6.60, 95% CI: −9.16 to −4.10), with marginal plots showing near-zero leaf area under extreme conditions. Moderate intensity increased leaf area substantially (Estimate = 1.26, 95% CI: −1.25 to 3.76), with marginal plots indicating values exceeding 600 units, suggesting that moderate disturbance may promote leaf growth. Timing effects were negative but weaker: early timing reduced leaf area (Estimate = −2.10, 95% CI: −4.71 to 0.48), while late and normal timings showed smaller reductions. Interaction terms indicated that the negative effect of extreme intensity was amplified under early timing (Estimate = −2.74, 95% CI: −5.66 to 0.20), whereas interactions with moderate intensity were positive but uncertain. Species-level variation was substantial (sd = 5.25, 95% CI: 2.42 to 11.78), and the shape parameter (0.21, 95% CI: 0.16 to 0.25) confirmed a highly right-skewed distribution.

**Figure 4:**
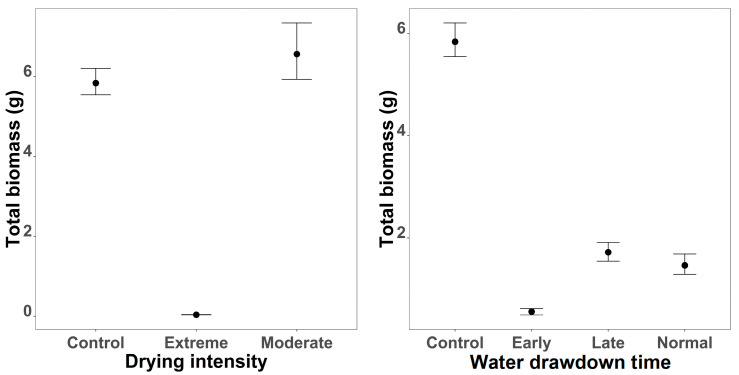
Effects of drying intensity (**left**) and water drawdown regime (**right**) on total biomass. Points represent posterior means, and error bars indicate 95% credible intervals based on 10,000 posterior draws of the final GLMM model.

Biomass was strongly affected by both timing and intensity (Figure 4). Control plots exhibited the highest estimated weight, whereas early timing resulted in a substantial reduction (Estimate = −2.46, 95% CI: −5.08 to 0.16). Late and normal timings also reduced biomass but to a lesser extent (Estimates = −1.31 and −1.45). Marginal plots showed mean biomass near 6 units under control, declining to approximately 0.5 units for early timing and around 1.5 units for late and normal timings. Intensity effects were even more pronounced: extreme intensity caused a dramatic decline (Estimate = −5.11, 95% CI: −7.63 to −2.61), with marginal plots indicating near-zero biomass under these conditions. Moderate intensity had little effect compared to control (Estimate = 0.06, 95% CI: −2.36 to 2.46). Species-level variation was considerable (sd = 2.33, 95% CI: 0.72 to 6.38), and the shape parameter (0.33, 95% CI: 0.27 to 0.41) confirmed a right-skewed distribution typical for biomass data.

#### 2.3.2. Species-Specific Responses to Drought

**Figure 5:**
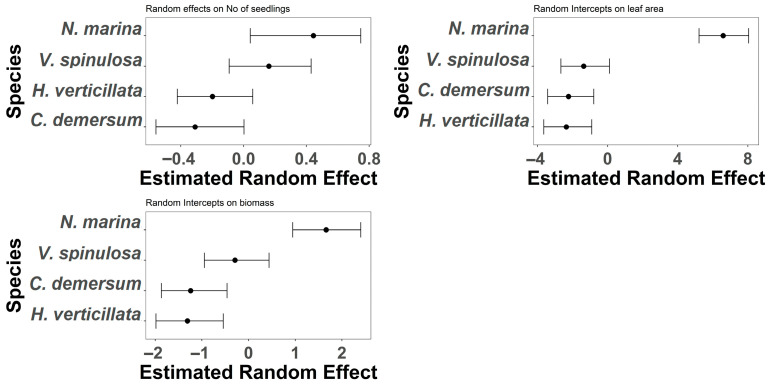
Random intercepts for species effects on biomass, leaf area, and seedling re-germination. Positive values indicate species with higher trait values than the overall mean, while negative values indicate lower values. *N. marina* consistently showed positive deviations, whereas *H. verticillata* and *C. demersum* were negative across most traits.

Species-level random effects revealed substantial variation in responses across all three traits (Figure 5). For biomass, *N. marina* exhibited the highest positive deviation from the overall mean, while *H. verticillata* and *C. demersum* showed negative deviations, indicating lower biomass under similar conditions. For leaf area, *N. marina* again had the largest positive effect, whereas *H. verticillata* and *C. demersum* were strongly negative, suggesting that leaf expansion is highly species-dependent. For seedling re-germination, *N. marina* and *V. spinulosa* showed positive deviations, while *H. verticillata* and *C. demersum* were negative, indicating that some species are more resilient to drought and disturbance than others. These patterns highlight the importance of species identity in mediating trait responses to timing and intensity treatments.

## 3. Discussion

The alignment of field observations and greenhouse experiments provides compelling evidence for how drought severity and timing shape submerged macrophyte communities. In the context of climate change, increasingly frequent and intense droughts are expected to accelerate macrophyte loss and reshape community composition in shallow lakes and floodplain wetlands [1,17]. Field surveys in West Dongting Lake documented a sharp decline in species richness and biomass from 2018 to 2023, concurrent with extreme drought conditions. Clonal species such as *Hydrilla verticillata*, *Ceratophyllum demersum*, and *Vallisneria spinulosa* nearly vanished from many sites, while *Najas marina* became dominant in areas previously devoid of submerged vegetation. These patterns support field studies showing that hydrological extremes shift dominance to species with robust seed banks [27,28].

Our greenhouse experiment confirmed and elucidated the mechanisms behind these field patterns. Extreme intensity drought nearly eliminated biomass, leaf area, and seedling emergence for clonal species. In stark contrast, *N. marina* exhibited measurable regeneration even under severe desiccation, reflecting its reproductive strategy centered on a persistent seed bank [29,30]. By understanding dormancy cycles and environmental cues, such resilience to drought can be explained [29,31,32]. Meanwhile, *Hydrilla* turions and rhizomes were highly vulnerable to drought, demonstrating significant mortality under drawdown conditions [33]. Moderate intensity drought resulted in nuanced outcomes. Leaf area increased significantly, aligning with the Intermediate Disturbance Hypothesis, which posits that moderate stress can reduce competition and stimulate growth [34,35,36]. However, intermediate stress did not enhance biomass or seedling counts, indicating that different traits respond distinctly. The timing of drought also mattered: early-season drawdowns were more detrimental to growth than later ones, consistent with vulnerability during early developmental stages [37,38].

### 3.1. Species Biology and Reproductive Strategy

The contrasting responses of clonal versus sexual species underscore reproductive mode as a key mechanism of resilience. Clonal species depend on structures like tubers, turions, and rhizomes that are highly sensitive to desiccation [32,33,39]. Although these perennial species are capable of sexual reproduction, they often rely primarily on clonal expansion to maintain spatial dominance under relatively stable hydrological conditions [6,40,41]. Our results suggest that this dependence on vegetative propagules became a major vulnerability during the extreme 2022 drawdown. Because the drought occurred during the peak growing and seed-setting season and water levels dropped to exceptionally low conditions, the desiccation stress likely exceeded the tolerance limits of these propagules before perennial populations could recover through alternative pathways [33,42].

In stark contrast, *Najas marina* exhibited a seed-bank-dependent, “rapid-turnover” reproductive strategy. Our greenhouse observations revealed that even when established from vegetative cuttings, *N. marina* transitioned to a reproductive stage and began fruiting within approximately one month, whereas its clonal counterparts remained strictly in a vegetative growth phase over the same period. This early transition to seed production may provide a form of “biological insurance”, allowing *N. marina* to secure recruitment potential before subsequent high-intensity disturbances. In addition, our greenhouse observations qualitatively indicated that *N. marina* tended to emerge later than the clonal species under the same conditions, a phenological pattern that may reduce early-season competition and favor establishment after disturbance [43]. Migratory waterbirds also aid seed dispersal in exposed sediments, enhancing recovery potential [31]. This mechanism is plausible in flyway wetlands such as Dongting Lake, although it was not directly tested in the present study. These findings reinforce resilience theory and alternate-stable-state models, in which soil seed banks can function as temporal buffers during extreme disturbances that would otherwise eliminate macrophyte cover [27,30,44].

At the landscape scale, these species-level differences were likely further filtered by spatial heterogeneity. Specifically, Qingshan Lake, an enclosed and relatively high-elevation area of West Dongting Lake, experienced a longer exposure period and a later re-flooding sequence during and after the 2022 extreme drought. Such conditions may have created a local establishment window for *N. marina* from the persistent seed bank while simultaneously suppressing the recovery of clonal dominants. Similar post-drought community reshuffling has been documented in other shallow lakes and floodplain wetlands, where extreme hydrological fluctuations favor opportunistic or seed-bank-mediated recovery pathways over established perennial dominance [45,46]. However, we cannot exclude the contribution of co-varying local factors (e.g., hydro-connectivity, water transparency, or microtopography) that may also influence establishment success and competitive release [47,48,49].

### 3.2. Management Implications Within the Climate Change Context

Projected increases in drought frequency and severity due to climate change—and flash droughts—will likely amplify these dynamics [50,51,52]. Extreme drought events risk repeated collapses of clonal dominance, whereas seed-bank–dependent species like *N. marina* may prevail under future climate extremes [53]. This shift does not necessarily imply a simple loss of vegetation, but it may alter community composition, regeneration pathways, and habitat structure in ways that affect long-term ecosystem functioning [54,55].

Where water-level regulation is feasible, management may benefit from adopting seasonally explicit ecological water-level “rule curves”, rather than relying solely on annual minima, to avoid rapid or prolonged exposure during key growth and propagule-formation periods [56,57]. Avoiding early or severe drawdowns appears essential to protect clonal species and preserve habitat complexity. Conversely, controlled moderate drawdowns timed after peak growth may help maintain species richness and diversity, consistent with strategies tested for *Hydrilla* control [33,34]. However, in our context, the primary objective is community persistence and recovery rather than suppression of a single target species; therefore, drawdown prescriptions should be evaluated against multi-species responses. Implementation should consider cumulative effects across multiple seasons, as repeated drawdowns can exhaust tuber banks [33].

Preserving and restoring soil seed banks is equally important for recovery. Although fire or desiccation can reduce seed viability over time, banks can remain functional for decades [29,30]. Fostering conditions that enhance seed bank inputs—such as periodic low-drawdowns timed for seed set—can strengthen resilience in the face of increasing hydroclimatic variability [58,59]. In addition, restoration studies often emphasize the importance of protecting remnant vegetated patches as propagule refugia and, where natural recovery is slow, applying targeted propagule augmentation (e.g., limited transplanting or seeding) to accelerate recolonization [60,61]. Such interventions should be applied cautiously and paired with monitoring to minimize unintended shifts in community composition.

### 3.3. Limitations and Future Directions

Although controlled greenhouse conditions helped isolate key variables, the field patterns observed after the 2022 extreme drought likely reflect additional processes operating in natural lake wetlands, including nutrient dynamics, sediment interactions, and competition [53,62,63].

Future studies should (i) extend simulations across repeated drought–rewetting cycles and explicitly vary drawdown timing, duration, and rate to test for cumulative and threshold responses [56,64]; (ii) quantify seed-bank density and viability under contrasting soil and sediment conditions to strengthen inference on recruitment potential and post-disturbance recovery pathways [29,65], and (iii) develop trait-based predictive models that link regeneration strategy, propagule type, and phenology to drought exposure and recovery under climate variability [33,39,42,66]. Finally, field or mesocosm trials that manipulate water levels in representative wetlands will be crucial for validating these greenhouse-based recommendations, particularly under projected climate extremes [56,67].

## 4. Materials and Methods

### 4.1. Study Area

Dongting Lake is located in the northeastern part of Hunan Province, within the middle and lower reaches of the Yangtze River in China (111°53′–113°08′ E, 28°44′–29°35′ N). The lake has a natural water surface area of approximately 2691 km^2^ and a volume of about 1.74 × 10^10^ m^3^. Due to its historical evolution and extensive sedimentation, Dongting Lake has been divided into three sub-lakes: West Dongting Lake, South Dongting Lake, and East Dongting Lake [68]. West Dongting Lake, situated in the western part of the system, is a typical river-connected lake that receives inflows from the Lishui and Yuan Rivers, as well as distributaries from Songzi, Taiping, and Ouchi [69]. It is the highest-elevation sub-basin within Dongting and exhibits strong seasonal hydrological fluctuations, with a characteristic transition from “lake during high water, floodplain during low water” [70]. Water levels begin rising in April, peak between July and September, and drop rapidly into a prolonged dry period during the winter [71].

**Figure 6:**
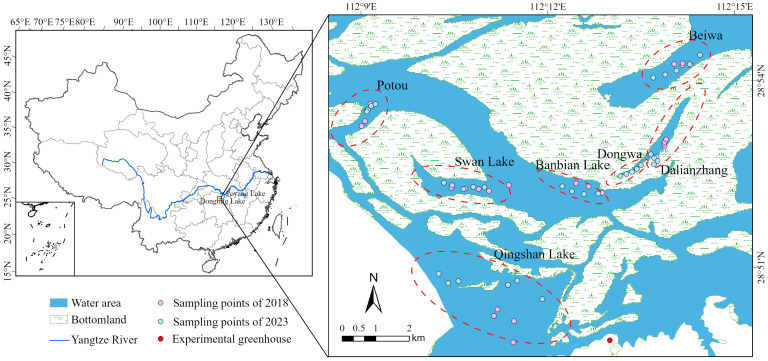
Location of study area and sampling design. The left panel shows the geographical context within China, indicating the positions of the Yangtze River, Poyang Lake, and Dongting Lake. The right panel illustrates the six sampling regions (Beiwa, Potou, Swan Lake, Banbian Lake, Dalianzhang, and Qingshan Lake) and all sampling points surveyed in 2018 and 2023, as well as the site of the subsequent greenhouse experiment.

The West Dongting Lake National Nature Reserve (28°47′–29°07′ N, 111°57′–112°17′ E; Figure 6) is a nationally designated wetland nature reserve and was listed as a Ramsar Wetland of International Importance in January 2002 [72]. The reserve harbors high biodiversity and provides essential habitats for submerged macrophytes, migratory birds, and aquatic fauna, making it a valuable site for both conservation and scientific research in subtropical inland wetlands [73]. The region has a mid-subtropical monsoon climate, characterized by distinct seasonal alternation between a wet summer and a dry winter [74]. While in recent years, the region has experienced increasingly frequent extreme climate events, such as prolonged droughts and erratic rainfall, resulting in intense water level fluctuations and earlier onset of the dry season [70]. These hydrological shifts have raised serious concerns about their potential impacts on the regeneration and persistence of submerged macrophyte communities [75].

### 4.2. Field Survey

Field surveys were conducted in August 2018 and August 2023, during the summer high-water season, in the West Dongting Lake wetland. Six representative sampling regions—Potou, Swan Lake, Beiwa, Dalianzhang, Qingshan Lake, and Banbian Lake—were selected for investigation (Figure 1). In each sub-region, five replicate sampling points (replicate plots) were established and distributed as evenly as possible using a transect-based method. Submerged macrophytes were collected using a custom-made iron rake (30 cm long × 10 cm wide), with each sampling plot covering an area of 0.3 m^2^. Sampling was conducted at water depths ranging from 0 to 4 m, and the geographic coordinates of each sampling points were recorded using a handheld GPS device (Garmin GPSMAP 63csx, Garmin Ltd., Schaffhausen, Switzerland).

All plant samples were thoroughly rinsed with clean water to remove sediment and debris, sorted by species, and placed into paper envelopes. The samples were then oven-dried at 80 °C for 72 h until a constant weight was reached. Dry biomass was measured using an electronic balance with a precision of 0.001 g, and the values were recorded accordingly.

### 4.3. Greenhouse Experiment

**Figure 7:**
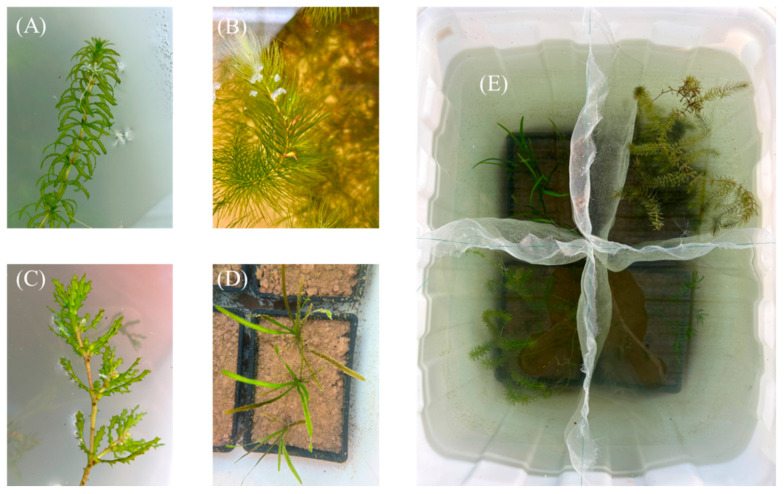
(**A**) represents *H. verticillata*, (**B**) represents *C. demersum*, (**C**) represents *N. marina*, and (**D**) represents *V. spinulosa*. Panel (**E**) shows all four submerged macrophyte species placed together in a single tank, separated by mesh to prevent physical entanglement.

This experiment was conducted from August 2024 to May 2025 in a greenhouse at the West Dongting Lake National Nature Reserve (28°49′36″ N, 112°12′11″ E). The greenhouse is located within the West Dongting Lake region, ensuring that ambient temperature and photoperiod broadly match local conditions, while allowing controlled manipulation of water drawdown treatments to simulate seasonal drought. Four typical and common submerged macrophyte species from Dongting Lake were selected for the experiment [76]: *H. verticillata*, *C. demersum*, *N. marina* and *V. spinulosa. H. verticillata* (Hydrocharitaceae; *Hydrilla*, Figure 7A) is a perennial submerged macrophyte that reproduces predominantly vegetatively. It also sets seed, but sexual reproduction contributes little to population expansion [77]. *C. demersum* (Ceratophyllaceae; *Ceratophyllum*, Figure 7B) is a perennial submerged macrophyte normally grow with the base of its stem buried in sandy or silty substrates. It is typically found floating in stagnant and slow-moving water [78]. The species pollinates underwater, can set seed, but typically regenerates via fragments [63]. *N. marina* (Hydrocharitaceae; *Najas*, Figure 7C) is an annual submerged macrophyte; it pollinates entirely underwater, and population renewal is driven mainly by seed production and a persistent soil seed bank [29,79]. *V. spinulosa* (Hydrocharitaceae; *Vallisneria*, Figure 7D) is a perennial, rosette-forming large submerged macrophyte [80]. It reproduces sexually and also clonally via stolons, with population expansion largely driven by clonal propagation [81]. Plant materials were procured in August 2024 from local suppliers of submerged macrophytes or collected from adjacent lakes (e.g., the Maoli Lake Wetland); their provenance and morphology were consistent with conspecifics from the Dongting Lake region and four pots (one for each species) were placed in a 200 L rectangular tank (83 × 60 × 55 cm), separated by mesh to prevent physical entanglement among plants (Figure 7E).

**Figure 8:**
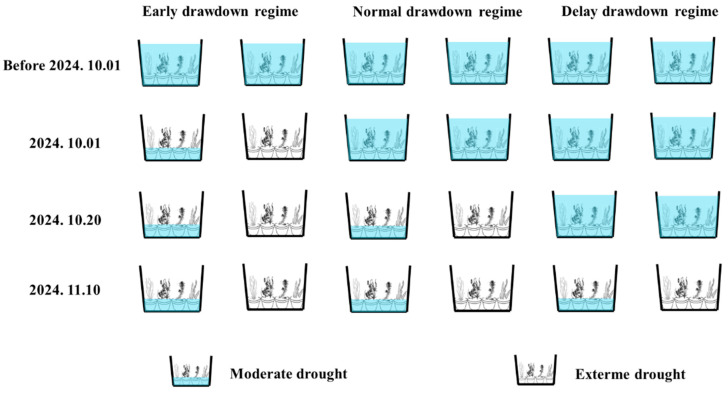
Schematic diagram of the controlled experiment design. Each tank contained four pots, each planted with a different submerged macrophyte species. Prior to treatment, uniform water level management was applied to ensure similar growth status across all plants. From left to right, every two columns represent one of the three drawdown regimes: early (initiated on 1 October), normal (20 October), and late (10 November). For each drawdown regime, four replicate tanks were established and subjected to either moderate or extreme drought treatments. All submerged macrophytes were harvested uniformly in May of the following year.

The experiment began on 12 August 2024. Planting substrate consisted of sediment collected from elevated areas adjacent to West Dongting Lake that have remained above the historical maximum inundation level for many years and therefore are unlikely to contain a persistent submerged macrophyte seed bank. Prior to the formal treatment phase, we applied a uniform water-level management regime to allow all submerged macrophyte seedlings to develop into fully formed individuals, a process lasting approximately 1.5 months. During this period, any underdeveloped or eliminated individuals were promptly replaced with healthy plants from a backup pool, ensuring that all submerged macrophytes exhibited similar growth status at the start of the formal experiment. The experiment followed a fully randomized two-factor design, with two factors: drawdown timing (early: 1 October; normal: 20 October; late: 10 November) and drought intensity (moderate: soil remains moist; extreme: soil completely dries and cracks). A control group without drawdown was also included. Each treatment had four replicates, totaling 28 tanks (Figure 8). After drawdown, no water was added during the winter, simulating natural exposure during the dry season. In early March 2025, all tanks were slowly rewatered to simulate the spring flooding process, and plant growth was subsequently monitored. The final harvest was conducted on 5 May 2025.

At harvest, sediments were washed with a high-pressure water jet to preserve intact roots and shoots. For each pot, the number of surviving individuals and their reproductive mode (sexual or asexual) were recorded. For *H. verticillata*, *C. demersum*, and *N. marina*, the three longest surviving individuals per pot were selected for stem/leaf length measurements, whereas all surviving individuals were scanned (Canon LiDE 300, Tokyo, Japan) to quantify total leaf area. For *V. spinulosa*, the three longest surviving individuals were selected for leaf-length measurements and were also scanned to estimate leaf area. Leaf area was calculated using Fiji (ImageJ2, version 2.16.0; National Institutes of Health, Bethesda, MD, USA) and divided by the number of individuals to obtain the mean leaf area per plant. Aboveground and belowground parts were separated, oven-dried at 80 °C for 72 h to a constant weight, and weighed to determine dry biomass using an electronic balance with a precision of 0.001 g. As *C. demersum* lacks true roots and thus belowground biomass was negligible and *H. verticillata* has negligible belowground biomass, total biomass was recorded for further analysis.

### 4.4. Data Analysis

Field survey data were visualized to illustrate the extensive decline of clonal species and the rapid spreading of *N. marina.* Stacked bar plots were generated to illustrate the average biomass of six submerged macrophyte species across six lakes and two survey years: 2018—a normal year, and 2023—after the extreme drought 2022.

All statistical analyses for the greenhouse experiment were conducted within a Bayesian framework using the R package brms [82], which provides flexible generalized linear mixed models (GLMMs). This approach was chosen because traditional ANOVA assumes balanced datasets, normality, and homoscedasticity, whereas GLMMs allow for non-normal distributions, hierarchical structures, and zero-inflation [83,84]. In addition, Bayesian approaches have the advantage of estimating parameter probabilities by controlling for the influence of noisy data and insufficient samples [85].

For each response variable—(i) total number of re-germinated seedlings, (ii) leaf area, and (iii) total biomass—we fitted full models including the main effects of drawdown timing and drought intensity, as well as their interaction. Species was included as a random effect to account for interspecific variability.

Count data (number of seedlings): Candidate link functions included Poisson, negative binomial, geometric, zero-inflated Poisson, and zero-inflated negative binomial. Model comparison indicated that the negative binomial distribution provided the best fit.

Continuous data (leaf area and biomass): We evaluated Gaussian, Student-t, log-normal, skewed normal, Gamma, and Weibull distributions. The Gamma distribution was selected as it best accommodated heavy tails and outliers.

Model selection followed a hierarchical approach: if the interaction term was not supported, we refitted a reduced model excluding the interaction and compared it to the full model using leave-one-out cross-validation (LOO). The model with the lowest expected log predictive density (ELPD) was retained as the best-fitting model.

Posterior estimates and credible intervals were derived from 4 chains of 10,000 iterations each, with convergence assessed via R-hat statistics (<1.01) and effective sample sizes. Model diagnostics included posterior predictive checks and residual plots to ensure adequate fit.

Leave-one-out cross-validation (LOO) was used to compare candidate models for biomass, leaf area, and seedling re-germination. For biomass, the generalized linear mixed model (GLMM) with a gamma distribution and log link provided the best predictive performance, and the model without interaction terms (including only main effects of drawdown timing and drought intensity) was selected. Similarly, for seedling re-germination, the negative binomial GLMM with main effects of timing and intensity was preferred. In contrast, for leaf area, the gamma GLMM that included an interaction between timing and intensity achieved superior predictive accuracy and was retained. All selected models showed satisfactory convergence (Rhat = 1.00 for all parameters) and adequate effective sample sizes.

## 5. Conclusions

This study demonstrates that extreme drought can restructure submerged macrophyte communities in large shallow lake wetlands by shifting dominant regeneration pathways. Field surveys following the 2022 drought in West Dongting Lake showed a marked decline of clonal-dominant perennials (e.g., *H. verticillata* and *V. spinulosa*), whereas the seed-bank–dependent annual *Najas marina* re-established successfully, particularly in the enclosed, high-elevation Qingshan Lake area. Greenhouse observations provide mechanistic support for this field pattern: *N. marina* rapidly transitioned to reproduction and likely benefited from seed-bank buffering, while clonal species remained reliant on vegetative growth and may be more vulnerable when severe desiccation impairs vegetative propagules.

Under increasing hydroclimatic extremes, sustaining submerged macrophyte diversity and habitat structure will likely require water-level management that is sensitive to both drawdown magnitude and seasonal timing. Where regulation is feasible, seasonally explicit ecological water-level targets that avoid rapid or prolonged exposure during key growth and propagule-formation periods may reduce collapse risk for clonal species. Long-term resilience also depends on safeguarding propagule sources, including persistent soil seed banks and remnant vegetated patches that function as local refugia. Where natural recovery is slow, carefully designed, small-scale reintroduction (e.g., transplanting or seeding), coupled with monitoring, may accelerate recolonization while minimizing unintended shifts in community composition. Together, our findings provide a field-grounded and mechanism-informed basis for anticipating and managing macrophyte recovery trajectories in drought-prone shallow lakes and floodplain wetlands.

## Data Availability

The data supporting the findings of this study are available from the corresponding authors upon reasonable request.
